# Collaborating with a robot biases human spatial attention

**DOI:** 10.1016/j.isci.2025.112791

**Published:** 2025-06-02

**Authors:** Giulia Scorza Azzarà, Joshua Zonca, Francesco Rea, Joo-Hyun Song, Alessandra Sciutti

**Affiliations:** 1 Robotics Brain and Cognitive Sciences Unit, Italian Institute of Technology, 16152 Genoa, GE, Italy; 2 Department of Computer Science, Bioengineering, Robotics and Systems Engineering, University of Genoa, 16145 Genoa, GE, Italy; 3 Cognitive Architecture for Collaborative Technologies (CONTACT) Unit, Italian Institute of Technology, 16152 Genoa, GE, Italy; 4 Department of Cognitive & Psychological Sciences, Brown University, Providence, RI 02912, USA

**Keywords:** Classification Description, Robotics, Social sciences

## Abstract

Advancements in collaborative technologies have increased the accessibility of human-robot interaction (HRI), yet a gap remains in understanding how HRI affects human systems. This work investigates how HRI influences human visual processing, specifically through the near-hand effect, a phenomenon where the proximity of one’s hand enhances spatial attention. Research has shown that this attentional priority can extend to another person’s hand during collaborative tasks, integrating it into an individual’s body schema. A critical question arises: can a robot’s anthropomorphic hand similarly bias human attentional priority? Our findings revealed that collaborative HRI facilitates target detection near the robot’s hand, an effect absent before the interaction. Additionally, we examined social and kinematic metrics that enhance this attentional shift by fostering joint body schema formation. These results highlight that HRI can shape human visual processing and body schema integration, offering insight into the interplay between our perceptual and cognitive systems and robotic collaborators.

## Highlights

• Collaborative interaction with a robot can reshape human spatial attention
• After interaction, the human brain treats the robot hand as part of its body schema
• Engagement in the task and proximity to human peripersonal space foster joint body schema
• Attributing human likeness and competence to the robot strengthens the joint body schema

## Graphical abstract

**Figure:**
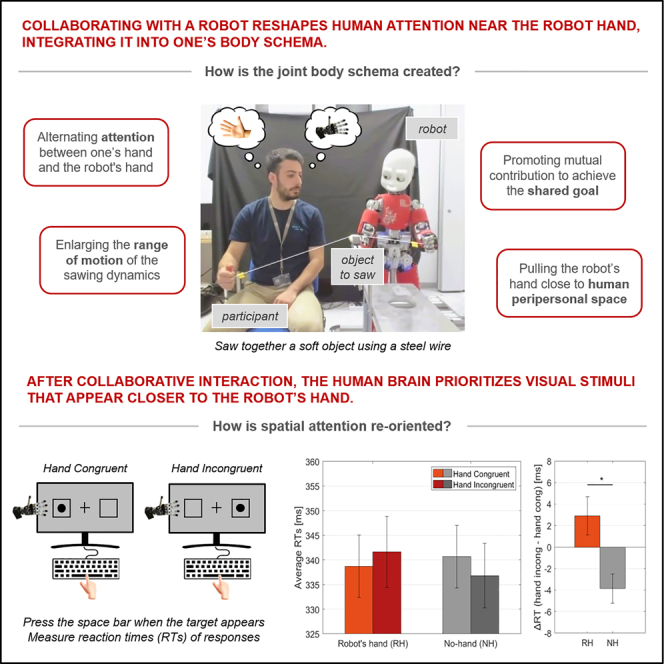

## Introduction

A distinctive characteristic of human beings is their ability to interact effectively, an ability that still greatly exceeds current collaborative technologies.^1^^,^^2^^,^^3^ Humans collaborate more frequently than any other primate species and often prefer joint activities over solo performance, even without apparent benefits.^4^ That is all the more remarkable considering that perceptual and motor abilities can differ significantly between individuals,^5^^,^^6^ making it essential to align our perceptions with those of our partners, understand their viewpoints, and adjust our actions accordingly.^7^^,^^8^ It has been proposed that social interaction represents the default mode in which the brain operates,^9^^,^^10^^,^^11^ suggesting that our ability to seamlessly coordinate with others stems from the brain’s evolution to support survival in social environments.^12^ Indeed, despite the complexity of understanding and predicting others’ actions, humans exhibit excellent capabilities at coordinating in time and space with other agents, as can be observed in everyday life activities,^13^ such as taking turns when talking or passing an object to a partner, and becomes central in contexts with high coordination demands, as sports,^14^ dance,^15^ and music.^16^

A large part of our skilled behavior, both when acting alone or with a partner, is enabled by a tight connection between our action and attention allocation processes. As a representative example, our visual system processes information differently based on its proximity to our hands.^17^^,^^18^^,^^19^^,^^20^ This near-hand effect is believed to arise from the hands’ behavioral relevance for action,^21^ leading to attentional prioritization and the engagement of bimodal neurons responsive to stimuli near the hands.^22^^,^^23^ This phenomenon is closely tied to the body schema, a structured model that maintains implicit spatial representations of the body’s dimensions and the relative positions of its segments.^24^ The body schema allows the brain to continuously update and adjust movements, integrating sensory input and motor commands, essential for interacting effectively with objects near the hands.^25^ Near-hand effect affects perception,^26^ memory,^27^ and semantic processing.^28^ It also influences attentional processing, leading to prolonged visual searches,^29^ delayed transitions between global and local aspects of hierarchical figures,^30^ and biased spatial attention toward the hands.^31^^,^^32^

Crucially for the effectiveness of collaborative interaction, the near-hand effect can extend to another person’s hand following collaborative tasks.^33^^,^^34^ Using a classical psychological paradigm, the Posner cueing task,^35^ Sun and colleagues found that enhanced target detection near one’s own hands could also occur near a partner’s hand, but only after a joint activity such as a joint sawing task. This suggests that individuals continuously adjust to their partner’s movements during collaborative tasks and integrate them into their body schema.^36^^,^^37^ During a collaboration, an interpersonal joint body schema would incorporate not only one’s physical dimensions and movements but also those of the collaborative partner.^38^ Although it is known that shared goals and intentions can facilitate this integration by aligning the attentional and motor systems of both parties,^39^^,^^40^ it remains unknown what factors influence the integration of others into an individual’s body schema.

The primary aim of this study is to explore the phenomenon that enables the integration of the near-hand effect with a partner’s hand, extending the body schema to include external (also non-human) agents. This research is vital for applications in rehabilitation, robotics, and augmented reality, where seamlessly incorporating others into the body schema could improve user interaction, coordination, and overall performance.

The experimental design revolves around a Posner cueing task, a well-established method for measuring visuospatial attention shifts, performed after collaborating with a humanoid robot. By analyzing reaction times to visual targets near different hands in individual settings or after a collaboration, we can infer to which extent the near-hand effect extends to the robotic partner. Additionally, collecting kinematic metrics and assessing the individual perception of the robot can provide insights into the phenomena that foster the formation of a joint human-robot body schema, by linking external behaviors to internal cognitive and perceptual processes. The robot, iCub,^41^ serves not just as a participant in the interaction but as a highly controllable and precise tool for experimental manipulation.^42^ The robot’s anthropomorphic design closely mimics the human form, allowing us to explore whether and how similar shapes can be integrated into the human body schema. Furthermore, the robot’s behavior and positioning can be precisely controlled and replicated across trials and participants, providing consistency and eliminating variability often present in human-human interactions.

The results of this study are expected to provide novel insights into the flexibility of the human body schema and the conditions under which external agents, such as robots, can be effectively integrated during collaborations.

## Results

**Figure 1:**
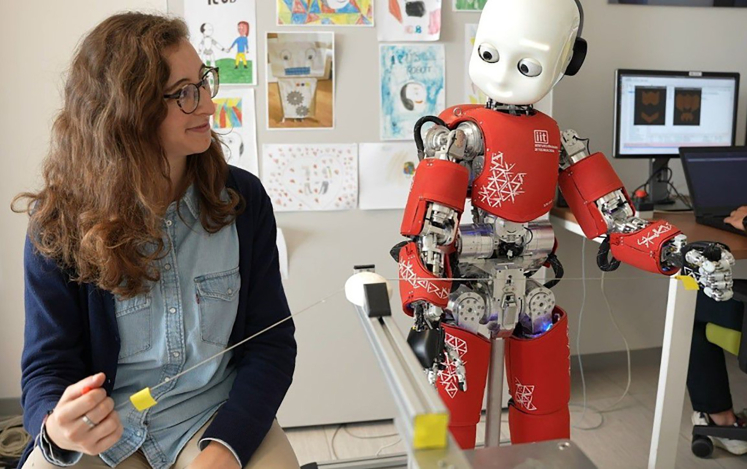
Human-robot joint sawing task The user and the robot hold one side of a steel wire each and take turns pulling to saw a soap bar. They create a cyclic movement by exchanging forces through the wire. The goal of the task is to maximize the indentation through the soap by developing an effective coordination strategy.

The goal of this study was to systematically investigate how a collaborative interaction with a humanoid robot could impact the development of a joint body schema, potentially influencing human visuospatial attention. To this end, we used the humanoid robot iCub to implement a human-robot collaboration consisting of a joint sawing task (Figure 1) inspired by previous work on collaborative physical joint action between humans.^34^ In particular, the goal of the cooperation was to maximize the indentation through a smooth object (i.e., a soap bar) using a non-rigid interface (i.e., a steel wire) by developing an effective coordination strategy. The participant and the robot held one side of the wire each and took turns pulling by exchanging forces through the wire. Thirty right-handed participants were involved in the study. Each participant did the task with either the right or the left hand. When the participant kept the handle in the right hand, the robot used its left hand and vice versa.

**Figure 2:**
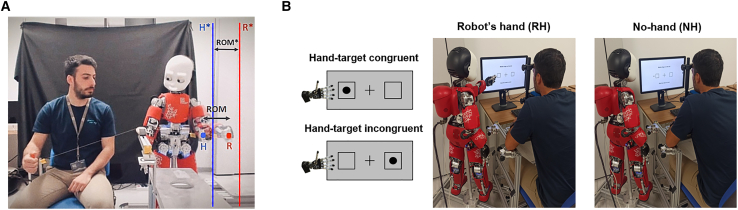
Design of the experiment: collaborative joint action and Posner cueing task (A) During the collaborative joint task, the human and the robot take turns pulling. The robot’s hand is programmed to move inside a predefined range of motion (ROM∗) among positions H∗ (human pulling, blue line) and R∗ (robot pulling, red line). The trajectory of the robot’s hand is highly affected by the force of the participant pulling, so iCub’s hand moves between positions H (human pulling, blue dot) and R (robot pulling, red dot). The robot’s hand typically overpasses the H∗ position when the participant is pulling, whereas it does not reach the R∗ position in the robot pulling turns. (B) The Posner cueing task was performed under two conditions: with the robot’s hand (RH) near the screen or with no hand (NH) near the screen as a control condition. Within the RH condition, trial classification was based on hand congruency, which prescribes how the robot’s hand and the visual target (black dot) are presented in each trial: hand-target congruent if the hand and target are on the same side; hand-target incongruent if they are on the opposite side.

The robot’s arms were compliant during the collaboration, meaning the participant pulling could perturb their position and motion. The selection of compliance has a dual purpose. Firstly, it ensures safety during interaction by preventing the robot from breaking. Secondly, it increases the perceived adaptability of the robot’s behavior, leading to more personalized interactions by following human-preferred movement. The iCub hand should move within a predefined range of motion (ROM∗) among two predefined positions, H∗ and R∗ (Figure 2A). In contrast, due to robot compliance, the actual trajectory depended on the human pull, with the robot’s hand often being pulled further toward the participants when they were pulling (actual position H in Figure 2A) and being stopped before reaching the final position when it was the robot’s turn to pull (actual position R in Figure 2A).

After the interaction, the participants performed a Posner cueing task,^35^ an exercise consisting of detecting visual targets appearing on a screen as rapidly as possible. We tested two experimental conditions for the Posner: robot’s hand near the screen (RH) and no hand near the screen (NH) during the task (Figure 2B). The experiment included four blocks, split between RH/NH conditions (2 blocks/condition), alternating in a pseudo-randomized order (see “procedure and design”). In RH blocks, trials were categorized based on whether the target appeared on the same side as the robot’s hand near the screen, i.e., hand congruent trial, or on the opposite side, i.e., hand incongruent trial (Figure 2B). In the NH condition, we refer to congruent and incongruent trials considering the target location next to where the hand would be in the RH condition, even though no hand is presented near the screen.

### Near-hand effect of the robot’s hand occurs after collaboration

We focused on participants’ reaction times (RTs) for target detection as the dependent measure in the analysis (see “statistical analysis”).

First, we tested for the presence of the classical effect of cue validity in the Posner cueing task. We ran a mixed-effect linear model with trial-by-trial reaction times as the dependent variable and cue validity as the independent factor. Results reveal a significant effect of cue validity (B=−0.04, β=−0.39, z=−12.90, p<0.001). Anyway, cue validity had no impact on the near-hand effect emergence.

Then, we computed the RTs difference between when a target appears on the opposite side of the robot hand (hand-incongruent trials) and when the hand is on the same side as the target (hand-congruent trials), as reported in Equation 1.

(Equation 1) $$\Delta RT=RT_{hand\_incongruent}-RT_{hand\_congruent}$$

We defined the near-hand effect (NHE) as the faster RTs in the Posner cueing task when a target appears near the robot hand (RH condition), compared to when the robot hand is not near the screen (NH condition), as reported in Equation 2.

(Equation 2) $$NHE=\Delta RT_{RH}-\Delta RT_{NH}$$

Specifically, we analyzed the interaction between hand congruency and hand presence that expresses the NHE, i.e., the hypothesized decrease in reaction times when (1) the hand is present on the screen and (2) its position is coherent with the target location in the Posner cueing task.

Thus, we tested our primary hypothesis on the emergence of the NHE by the robot’s hand after the collaborative HRI. To this aim, we ran a mixed-effect model with trial-by-trial reaction times as the dependent variable and hand congruency, hand presence, and their interaction as independent variables. Findings suggest a significant interaction between hand congruency and hand presence (B=−0.01, β=−0.07, z=2.15, p=0.032), which indicates the NHE presence.

**Figure 3:**
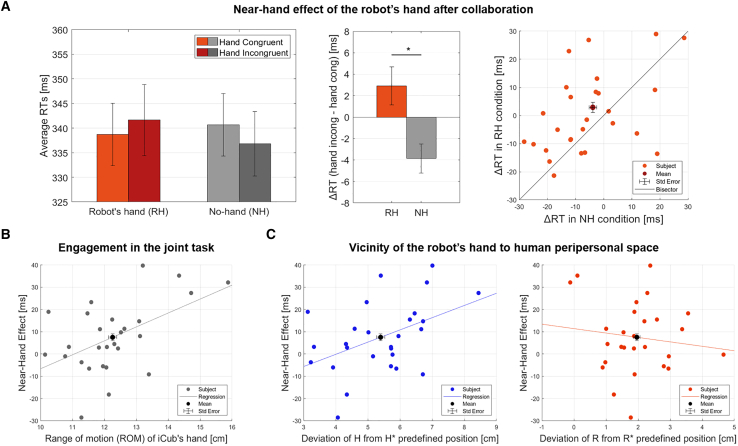
Results of the near-hand effect after collaboration, engagement in the joint task, and vicinity to human peripersonal space (A) The first bar plot (left) shows the average RTs across conditions. The red bars refer to the RH condition, and the gray ones refer to the NH condition, with light bars representing hand-congruent trials and dark bars representing hand-incongruent trials. The error bars represent the between-subjects SE of the mean. This plot highlights the key finding of our study: faster RTs in the hand-congruent RH condition after collaboration, leading to near-hand effect (NHE) of the robot’s hand. The second bar plot (middle) displays the NHE observed during the Posner task after the human-robot collaboration. Each bar is computed as the difference between the average RTs in hand incongruent minus hand congruent trials (see Equation 1). The error bars represent the between-subjects SE of the mean. Asterisk refers to mixed-effects model results, where ∗p<0.05. There is a significant difference between $\Delta RT_{RH}$ (red bar) and $\Delta RT_{NH}$ (gray bar), meaning the presence of the robot’s hand speeds up participants’ mean RTs for target detection. The scatterplot (right) shows that the emerging NHE is consistent across participants as most dots, each representing a subject, lie above the bisector. (B) A positive correlation exists between the NHE and the range of motion (ROM) of iCub’s hand. A high ROM may prescribe mutual engagement in the collaboration, so we find higher NHE when both parties are actively involved in the task. (C) The scatterplots display the positive correlation between the NHE and the deviation of position H from H∗ (left), which indicates a higher vicinity of the robot’s hand toward the human peripersonal space (PPS), and the negative correlation between the NHE and the deviation of position R from R∗ (right). Closeness to human PPS might increase the inclusion of the robot’s hand in the human body schema, facilitating the forming of an interpersonal joint body schema.

This result was corroborated by a one-sample Wilcoxon signed-rank test on the distribution of the NHE score, which confirmed the presence of a significant effect ($H_{0}$: NHE = 0, Z = 2.17, *p* = 0.030, Rank biserial correlation (Rbc) = 0.45). Results revealed that following the human-robot collaboration, the robot’s hand triggers the NHE effect on the Posner task. The observed NHE is consistent across participants, as most exhibit shorter mean RTs when the robot’s hand is present near the screen compared to when no hand is there (Figure 3A).

### Engagement in the joint task and vicinity of the robot’s hand to human peripersonal space strengthen the near-hand effect

Kinematic data were collected through sensors on the robot’s arms. Data analysis allowed us to explore the engagement of the parties in the joint task and how human behaviors influence the robot’s movement. Our hypothesis is that these kinematic factors may directly influence the integration of the robot’s hand into the human body schema.

We used correlations for statistical analysis to compare the NHE of the participants with the kinematic characteristics of our interest (introduced in Figure 2A), i.e., the range of motion (ROM) of the iCub’s hand and the deviation of its position toward the human peripersonal space (PPS), measured as the displacement of the H position from the predefined H∗ position. We excluded three participants from the analysis due to errors in saving of the kinematics features. Therefore, analyses refer to twenty-seven subjects.

The first test analyzed the correlation between the NHE and the ROM amplitude. Results found a positive correlation (Pearson’s r=0.511, p=0.006), suggesting that a wide ROM is associated with an increasing NHE (Figure 3B). The ROM size prescribes the active involvement of the parties in the collaboration since it is necessary that both maximize their effort in pulling (and letting the partner pull) to increase the ROM. We hypothesize that mutual engagement in the joint task is one of the components potentially facilitating the formation of a joint body schema during the interaction.

In the second test, we investigated the correlation between the NHE and the deviation of H position from the H∗ predefined position (marked in blue in Figure 2A). The analysis found a positive correlation (Pearson’s r=0.451, p=0.018). The vicinity of the robot’s hand to the human PPS correlates with the increasing attentional shift toward the robot’s hand, i.e., with the NHE (Figure 3C, left). We hypothesize that higher proximity of the robot’s hand to the human PPS could determine to what extent we incorporate the robot’s hand in our body schema, thus representing another factor fostering the emergence of a joint body schema.

Finally, we considered the deviation of R position from the R∗ predefined position (marked in red in Figure 2A). We found no significant correlations with the NHE (Pearson’s r = 0.130, *p* = 0.519). Results suggest the displacement of the robot’s hand from the R∗ position, which is the farthest from human PPS, has no direct impact on the NHE emergence (Figure 3C, right).

### Attribution of anthropomorphism, competence, and mind to the robot positively impacts joint body schema emergence

After the collaborative task, we asked participants to complete questionnaires on their impressions and perceptions of the robot. Questionnaires highlight the role of individual variables in shaping the interaction. By linking these metrics with behavioral results, we can reveal how perceived robot characteristics influence attentional priorities.

We proposed three questionnaires: the Godspeed Questionnaire Series (GQS),^43^ the Robotic Social Attributes Scale (RoSAS),^44^ and the Mind Perception.^45^ The GQS (5-point Likert) measures five critical concepts in HRI, expressed by five scale dimensions: anthropomorphism, animacy, likability, perceived intelligence, and perceived safety, to assess the user perception of the robot. The RoSAS (9-point Likert) is an 18-item scale to measure people’s judgments of the social attributes of robots, expressed by three underlying 6-item scale dimensions: warmth, competence, and discomfort. Mind Perception is the attribution of psychological states, such as thoughts, emotions, and intentions to humans, animals, or even inanimate objects such as robots and virtual characters. Mind Perception (7-point Likert) has two primary dimensions: experience (e.g., the ability to feel pain) and agency (e.g., the ability to self-control), assessed with four items each.

Initially, we computed Cronbach’s alpha^46^ to check internal consistency, which is a way to assess the scales’ reliability by measuring how closely related a set of items is as a group. The computed alpha was always greater than or equal to 0.70, an acceptable threshold in most social science research situations. Table 1 reports the correlations of the NHE with the GQS, RoSAS, and Mind Perception scales. Results found significant positive correlations with three of the GQS scales, i.e., anthropomorphism (Pearson’s r = 0.380, *p* = 0.038), animacy (Pearson’s r = 0.406, *p* = 0.026), and likability (Pearson’s r = 0.458, *p* = 0.011). Moreover, a positive correlation was found with the competence dimension of the RoSAS (Pearson’s r = 0.468, *p* = 0.009). Finally, the NHE is positively correlated with the experience dimension of the Mind Perception (Pearson’s r = 0.367, *p* = 0.046).

**Table 1:** Correlations between the near-hand effect (NHE) and the questionnaires: GQS, RoSAS, and Mind Perception

		Godspeed Questionnaire Series					Robotic Social Attributes Scale			Mind Perception	
		Anthrop*.*	Animacy	Likability	Intelligence	Safety	Warmth	Competence	Discomfort	Experience	Agency
NHE	*Pearson’s r*	**0.380∗**	**0.406∗**	**0.458∗**	0.337	0.246	0.286	**0.468∗∗**	0.135	**0.367∗**	0.285
	*p value*	**0.038**	**0.026**	**0.011**	0.068	0.191	0.126	**0.009**	0.478	**0.046**	0.126

The columns report, respectively, the five GQS scales, the three RoSAS scales, and the two Mind Perception dimensions. Significant correlations with the NHE are marked in bold. In particular, positive correlations between the NHE and the scales of the GQS (anthropomorphism, animacy, and likability) underline that the robot’s perception as a human-like animated agent impacts our attention allocation. Moreover, the positive correlation with the competence scale of the RoSAS highlights how attributing skills to the robot impacts the interaction. Finally, the positive correlation with the experience dimension of Mind Perception highlights that the perceived robot’s ability “to feel” influences the emergence of an attentional priority.

These findings highlight that the more human-like and competent the robot is perceived to be, the more impact its hand has after collaboration on human attentional prioritization. It appears then that the degree of animacy and mind attributed to an agent can also influence the extent to which a joint body schema is formed with it.

### Is the mere presence of the robot’s hand enough to trigger the near-hand effect?

What remained unclear was whether there exist an *a priori* effect of the robot’s hand without interaction or if collaborative interaction was the crucial point to the emergence of the NHE. To answer this question, we conducted a control study assuming two possible scenarios.

If we consider the robot’s hand as another person’s hand, we would expect the absence of spatial priority (i.e., the NHE) near the iCub’s hand without interaction. In a similar experiment, Sun and Thomas^34^ showed that people detected targets near their hand faster, but no similar attentional bias was observed for targets near a friend’s hand. Their results suggest that spatial attention is prioritized near one’s hand but is not automatically directed toward another’s hand.

Differently, if the robot’s hand is perceived as a fake hand, we could expect to observe spatial prioritization near the robot’s hand without interaction. In another study, Reed and colleagues demonstrated that when participants wore a rubber glove matching a glove on a fake hand displayed in front of them, they detected targets near the fake hand more quickly than those located further away.^31^

These findings suggest that people may prioritize space near a fake hand because they represent it in multisensory areas. The visual information about the hand position provided by the fake hand was sufficient to facilitate responses to the target appearing nearby. However, although the visual information available in the friend’s hand condition was quite similar to that in the fake hand condition, the experiment’s results suggest that the visual system treats a real person’s hand differently than a fake rubber hand. Specifically, no attentional bias was observed toward the real person’s hand, indicating a distinct processing mechanism for real versus artificial hands.

**Figure 4:**
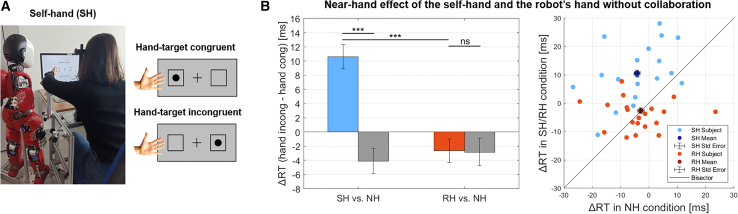
Design of the Posner cueing task (SH condition) and results of the near-hand effect without collaboration (A) The Posner cueing task was performed under three conditions. In addition to the RH and NH conditions (see Figure 2B), participants did the task with their hand, i.e., self-hand (SH), near the screen. Both conditions with the hand presence (human or robot) were compared to NH, and for both conditions, trial classification was based on hand congruency: hand-target congruent if the hand and target are on the same side; hand-target incongruent if they are on opposite sides. (B) The bar plot (left) displays the near-hand effect (NHE) observed during the Posner task without collaboration. Each bar is computed as the difference between the average RTs of hand incongruent minus hand congruent trials. The error bars show the between-subjects standard error of the means. Asterisk refers to mixed-effects model results, where ∗∗∗p<0.001 and “ns” means *not significant*. A significant difference exists between the $\Delta RT_{SH}$ (blue bar) and the $\Delta RT_{NH}$ (gray bar) but not between the $\Delta RT_{RH}$ (red bar) and the $\Delta RT_{RN}$ (gray bar). These findings suggest that the human self-hand speeds up RTs for target detection, but the robot’s hand does not. The scatterplot (right) shows that the NHE induced by the presence of the self-hand near the screen is consistent across participants. Most blue dots lie above the bisector, meaning participants’ average RTs are shorter in SH than in NH conditions. Differently, the robotic condition reveals poor consistency of the NHE across subjects. The red dots are distributed along the bisector, underlining no significant difference between RH and NH conditions. (See Figure S1 in the supplemental information for additional bar plots showing the average RTs across conditions).

Therefore, we ran a control study to assess whether the mere presence of the iCub’s hand, without the need for previous collaborative interaction, could induce a NHE while confirming that our setup could reliably produce a NHE with the participant’s hand, as observed in numerous studies.^29^^,^^31^^,^^34^ Twenty-two right-handed participants performed the Posner task in three conditions: with their hand near the screen (self-hand, SH), with the robot’s hand near the screen (RH), and with no hand near the screen (NH) as a control condition. The SH and RH conditions were compared to the NH control condition. The experiment included four sessions. Each session consisted of four blocks: two blocks with a hand near the screen (human or robot) alternated with two NH blocks (see “procedure and design”). In SH and RH blocks, trials were categorized based on whether the target appeared on the same side as the hand near the screen (either human or robot), i.e., hand congruent or on the opposite side, i.e., hand incongruent (see Figure 2B for RH/NH condition; Figure 4A for SH condition).

### No effect of the robot’s hand without collaboration

This control study revealed the absence of NHE near the robot hand without prior interaction. This is consistent with the results obtained in human studies, where no NHE appeared near a friend’s hand prior to collaboration.^34^

We recall that participants’ reaction times for target detection were the dependent measure for our analysis. As in the principal study, we first confirmed the presence of a cue validity effect in the Posner cueing task (B=−0.04, β=−0.37, z=−8.97, p<0.001). Again, cue validity had no impact on the NHE emergence.

Then, we tested our main hypotheses on the absence of the NHE before the collaborative HRI. We ran a mixed-effect linear model with trial-by-trial reaction times as dependent variables, hand congruency, hand presence, hand identity (SH, RH), and their interactions as independent factors. Results revealed a significant interaction between hand congruency and hand presence ($\chi^{2}=17.47$, p<0.001) and a significant three-way interaction between hand congruency, hand presence, and hand identity ($\chi^{2}=18.18$, p<0.001). Fixed effects results showed that the NHE (hand congruency ∗ hand presence interaction) was present with the self-hand on the screen (B=−0.01, β=−0.15, z=−6.75, p<0.001) but absent with the robot’s hand on the screen (B=−0.00, β=−0.00, z=−0.13, p=0.896) (Figure 4B, left). A control model testing for a potential effect of hand side (left, right) on the NHE revealed no significant three-way interaction between hand congruency, hand presence, and hand side ($\chi^{2}=0.27$, p=0.606).

To corroborate the models’ results, we ran a one-sample Wilcoxon signed-rank test on the individual NHE scores in both hand identity conditions (${\text{NHE}}_{S}$ and ${\text{NHE}}_{R}$, see “statistical analysis”). Findings confirmed that the NHE was significantly different from 0 in the self-hand condition ($H_{0}$: ${\text{NHE}}_{S}$ = 0, Z = 3.95, p < 0.001, Rbc = 0.98), but it was not in the robot’s hand condition ($H_{0}$: ${\text{NHE}}_{R}$ = 0, Z = −0.16, *p* = 0.876, Rbc = −0.04). Furthermore, we used a Wilcoxon signed-rank test to compare directly ${\text{NHE}}_{S}$ and ${\text{NHE}}_{R}$. Results revealed a significant difference in the two hand identity conditions (Z = 3.35, p < 0.001, Rbc = 0.84). Findings suggest that the self-hand near the screen speeds up participants’ mean RTs for target detection; on the contrary, the robot’s hand does not necessarily direct human attention toward itself. The observed NHE is consistent across participants in the SH condition, but it is not in the RH condition (Figure 4B, right).

Observing our results, it is possible to notice that there is a significant difference in the NHE magnitude between the SH condition in the control study (Figure 4B, left) and the RH condition in the main experiment (Figure 3A, middle). This difference is reasonable considering that there exists an automatic prioritization of space near one’s own hand in attentional processing, whereas the robot’s hand requires integrating an external agent’s hand into one’s own body representation.

## Discussion

This research investigates how collaborative HRI impacts human visuospatial attention and body schema integration, highlighting the deep connection between human perceptual and cognitive systems and interactions with social robots.

In a series of experiments, we implemented a human-robot collaboration consisting of a joint sawing task. Following the collaboration, we used a classical Posner cueing task to measure the near-hand effect, a visual phenomenon where spatial attention is prioritized to the space near the hands, leading to faster target detection close to one’s own hand. Our goal was to experimentally measure the impact of human-robot collaboration on the human ability to reorient visual attention in space, particularly near the robot’s hand. This attentive effect was observed to extend to another person’s hand following shared collaborative tasks.^34^

Our findings demonstrate for the first time that a brief HRI, such as a joint sawing task, facilitates the detection of visual targets near the robot’s hand by reorienting human visuospatial attention to the space near the robot’s hand, an effect that did not exist before the joint action. Our results expand the research conducted with artificial objects, such as fake human-like hands,^47^^,^^48^ and other persons’ hands,^34^^,^^49^ demonstrating that an anthropomorphic robot hand, as a friend’s human hand, is not per se sufficient to shift human visuospatial attention toward itself. Attention is prioritized to the space near another’s hand (human or robot) only after the latter becomes relevant to accomplish a shared goal. Nonetheless, one’s own hand is likely to always exert a stronger attentional bias than another’s hand because it does not require body schema integration.

We hypothesized that the emergence of the near-hand effect following human-robot cooperation is strictly related to the inclusion of the robot’s hand into the human body schema, with a potential for extending the own body schema to include also the robotic partner’s hand, thus forming an interpersonal joint body schema. This interpretation takes inspiration from motor and social dynamics that develop in human interactions. Indeed, while interacting, humans continuously adjust their body schema to incorporate their partner’s kinematics, forming an interpersonal body schema that affects the partner’s attention allocation.^36^ When participants have a reason to incorporate a representation of another’s hands into their body schema, their visual systems show altered processing near these hands. The implications are noteworthy, suggesting a shared cognitive response to anthropomorphic features in both human and robotic entities.

To support this hypothesis, we identified social attribution and motor coordination metrics that could enhance this attentional prioritization by fostering the creation of a human-robot joint body schema. Collaboratively alternating attention and action between the own and robot’s hands became crucial for creating a boundary to achieve the shared goal.^40^ This repetitive dynamic enables a cyclical exchange of forces that highlights the human-robot collaboration, recalling cooperative tasks typically observed among human dyads.^50^^,^^51^ Moreover, the vicinity of the robot’s hand to the humans’ peripersonal space could determine to what extent they incorporate the robot’s hand in their body schema.^52^ Thus, the kinematic features highlight the importance of creating cyclic dynamics, enabling force exchange and active collaboration while sharing goals and effort. In addition, measurements of users’ perception of the robot (GQS scales^43^), its social attributes (RoSAS scales^44^), and its perceived ability to “feel” (Mind Perception^45^) proved that both perceived skills and human likeness impact attention allocation during interaction development.^53^ The robot’s competence increased its perceived skills, making it appear an intentional agent, whereas the human-like appearance and motion enhanced interaction effectiveness and improved collaborative performance.^54^

While the fixed behavior of the robot allowed us to precisely control and quantitatively assess the dynamics of the interaction, including the manipulation of the kinematics features, it restricts the potential for the creation of natural synchronization strategies during the interaction. Also, it precludes the customization of stimulus responses to emulate human-like behaviors, such as adjusting the movement velocity or miming the human action to reach smoothness, which works as bonding creation in human-human interactions.^55^ A crucial aspect of interactions concerns adaptation, a fundamental skill observed in (biological) cognitive agents, evident both in their behavior and physiological responses. Adaptability is essential for implementing artificial cognitive agents,^56^^,^^57^ enabling them to integrate into new environments, navigate changes in their surroundings, and establish the groundwork for a sophisticated, human-like exchange with other agents.^58^

Future research should exploit the adaptability of the iCub robot to personalize the collaborative task by exhibiting different levels of social intelligence^59^ and dynamic capabilities. The robot should adapt its behavior to that of the partner it faces to explore the underlying neural and cognitive mechanisms that might foster the formation of an interpersonal joint body schema (e.g., vision, touch, proprioception, etc.). Additionally, investigating how various types of robots (e.g., varying degrees of anthropomorphism, functionality, and interactive capabilities) affect the formation of this joint body schema could provide deeper insights, as we already proved how the perceived human likeness and competence impact the near-hand effect emergence. In particular, we aim to investigate whether interaction alone, regardless of the tool used, modulates attentional allocation. For instance, in future studies, we plan to test whether engaging in joint action with a non-anthropomorphic object, such as a wooden stick, would be sufficient to influence attentional processing.

In conclusion, advancements in collaborative technologies have improved the accessibility of social robots as counterparts in interactions.^60^^,^^61^ Thus, developing robots that can interact and collaborate efficiently and naturally with humans has become increasingly important.^62^ By understanding how and when a robot’s component can be integrated into the human body schema, designers can create more intuitive and effective interfaces, particularly in collaborative settings where humans and robots must work closely together. The knowledge gained from this research has significant implications for the design and deployment of robotic systems in human environments and could pave the way for advances in various fields, from prosthetics and rehabilitation to human-robot teaming in industrial and service environments.

### Limitations of the study

This study offers important evidence that collaborative interaction with a humanoid robot can modulate human visuospatial attention and contribute to joint body schema formation. However, some limitations should be considered when interpreting the findings. The robot’s behavior was pre-programmed and not adaptive, which, while ensuring experimental control, may not fully reflect the dynamic nature of real-world interactions. The human-robot interaction involved a single form of collaboration (i.e., the joint sawing task) which may limit the generalizability of the observed effects to other types of joint action. Additionally, only one robot platform with a specific anthropomorphic design (i.e., the iCub) was employed, which may influence the extent of human attentional integration. Finally, although subjective ratings of the robot’s attributes provided valuable insight into the social-cognitive mechanisms involved, they may be susceptible to individual interpretation biases. Despite these limitations, the controlled setup allowed for robust identification of the near-hand effect and highlighted the potential of collaborative human-robot interaction to influence fundamental cognitive processes.

## Resource availability

### Lead contact

Requests for further information and resources should be directed to and will be fulfilled by the lead contact, Giulia Scorza Azzarà (giulia.scorza@iit.it).

### Materials availability

This study did not generate new materials.

### Data and code availability

• Datasets supporting analyses and figures included in the current study are available in a dedicated OSF repository. Accession numbers are listed in the key resources table.
• Codes used to analyze the raw data, generate figures, and support results are available in a dedicated OSF repository. Accession numbers are listed in the key resources table.
• Any additional information required to reanalyze the data reported in this paper is available from the lead contact upon request.

## Acknowledgments

This work has been supported by a Starting Grant from the 10.13039/501100000781European Research Council (10.13039/100017325ERC) under the European Union’s H2020 research and innovation programme. G.A. No 804388, wHiSPER. We also acknowledge the support from the 10.13039/100000001National Science Foundation (NSF) BCS 2043328.

## Author contributions

All authors contributed to the design of the experimental protocol. G.S.A. programmed the experimental task with the support of J.Z. and F.R. G.S.A. carried out the experiments and the data analysis. Statistical analyses were supervised by J.Z. All authors provided critical feedback and helped shape the research, discuss the results, and write the final manuscript.

## Declaration of interests

The authors declare no competing interests.

## STAR★Methods

### Key resources table

**Table:** 

REAGENT or RESOURCE	SOURCE	IDENTIFIER
**Deposited data**		

Raw data and processed datasets	Open Science Framework	https://osf.io/gve6w/files/osfstorage

**Software and algorithms**		

Analysis and figure codes	Open Science Framework	https://osf.io/gve6w/files/osfstorage
YARP code	YARP: Yet Another Robot Platform	https://www.yarp.it/latest/

**Other**		

iCub humanoid robot	Italian Institute of Technology	https://icub.iit.it/

### Experimental model and study participant details

Fifty-two right-handed participants (32 females, 20 males) with normal or corrected-to-normal vision participated in our experiments. In particular, thirty performed the principal study (17 females, 13 males; mean age = 28.87 years; std = 9.38 years), including the collaborative joint action with the robot and the Posner cueing task, and twenty-two took part in the control study (15 females, 7 males; mean age = 26.05 years; std = 5.21 years) consisting only of Posner cueing task. Participants were all naive to the purpose of the study.

The Regional Ethics Committee approved the experimental protocol to protect human participants in research; all participants provided written informed consent before starting the experiment and received a refund after their participation.

### Method details

#### Apparatus

For the joint action, the experimental setup included a steel wire with two handles (held respectively by the participant and the robot), a new bar of soap for each participant, a rod to support the soap halfway between the participant and the robot, and a video camera to record the interaction. Each participant did the task either with the right or the left hand, maintaining consistency between the sawing action and the Posner task. Thus, if a person cut the soap holding the wire in the right hand, the same hand should be used to press the space bar to respond to visual stimuli; in this way, the robot used the left hand to cut the soap and put the same hand close to the screen in the RH condition of the Posner (see Figure S2A). When the setup was mirrored, the participant used the left hand to cut the soap and detect visual targets, whereas the robot used the right hand throughout the experiment (see Figure S2B).

For the Posner task, the participant and the robot were sitting close to each other. All visual stimuli were displayed in black on a light grey background on a monitor with a refresh rate of 60 Hz and a resolution of 1024 × 768 pixels. The experiment was coded in MATLAB using the PsychToolbox extension.^63^^,^^64^ A chin rest maintained a constant viewing distance from the screen (d=50cm). Participants responded by pressing the space bar on the keyboard. When instructed to place a hand near the screen, they rested their forearms on the support to minimize prolonged hand and arm extension discomfort. If the robot’s hand was placed near the screen, participants could rest the hand that was not used for responding to visual stimuli.

#### Procedure and design

In our principal study, participants collaborated with the iCub to perform a physical joint action. Since there was no training, before starting the interaction, we presented participants with a short tutorial video (30 seconds) showing a person performing the task with the iCub. At the beginning of the interaction, the iCub introduced itself and briefly repeated the goal of the task, which the participants had already been instructed about by the experimenter. At the end of the 4-minute cutting task, iCub thanked and greeted the participant.

After the interaction, the participants performed the Posner cueing task^35^ in two conditions: with the robot’s hand (RH) near the screen and with no hand (NH) near the screen (see Figure 2B). The experiment included one session consisting of four blocks (60 trials/block): two blocks with the robot’s hand near the screen (RH) and two blocks with no hand near the screen (NH). The RH blocks alternated with NH blocks. The block order was pseudo-randomized. The first two blocks must include one RH and one NH in random order, and the same for the last two. In RH blocks, trials were categorized based on whether the target appeared on the same side as the robot’s hand near the screen (i.e., hand-target congruent) or on the opposite side (i.e., hand-target incongruent).

The design of the Posner is structured as follows. In each trial, two empty squares (3.4 °) were positioned at 7.4 ° on either side of a central fixation cross (3.4 °). After a random delay of 1500–3000 ms, one square was cued by doubling the thickness of its border for 200 ms, and then a target, presented as a black dot (2.2 °), appeared. Participants had to press the space bar on the keyboard as soon as they saw the target. If the target appeared in the cued square, it was classified as a valid trial (see Figure S3A); if it appeared in the uncued square, it was classified as an invalid trial (see Figure S3B). Occasionally, the square remained cued for 2000 ms without the target appearing; these were the catch trials used to check response accuracy (see Figure S3C). In these cases, participants had to wait for the subsequent trial without pressing any keys. Each block included 70% valid trials (i.e., 42 trials), 20% invalid trials (i.e., 12 trials), and 10% catch trials (i.e., 6 trials) presented in random order, as done in.^34^

In the control study, participants performed the Posner task in three conditions: with their own hand near the screen (self-hand, SH), with the robot’s hand near the screen (RH), and with no hand near the screen (NH) as a control condition. The SH and RH conditions were compared to the NH condition (Figures 2B and 4A). The experiment included four sessions to test all combinations based on the conditions (RH vs. NH, SH vs. NH) and the hand used by the participants (right or left) in a randomized and counterbalanced order. Each session consisted of four blocks (60 trials/block): two blocks with a hand near the screen (self or robot) and two blocks with no hand near the screen. In each session, the SH and RH blocks alternated with NH blocks. The block order was pseudo-randomized. The first two blocks must include one SH/RH and one NH in random order, and the same for the last two. In SH and RH blocks, trials were categorized based on whether the target appeared on the same side as the hand near the screen (either self or robot) (i.e., hand-target congruent) or on opposite sides (i.e., hand-target incongruent).

### Quantification and statistical analysis

#### Statistical analysis

The analysis focused on participants’ reaction times (RTs) for target detection as a dependent measure. We excluded RTs outliers, discarding responses faster than 200 ms to eliminate anticipatory reactions^65^^,^^66^ and slower than 1000 ms, coming from low attention. In the principal study, we excluded 4.8% of trials as they fell outside the 200–1000 ms window. The error rate for participants in catch trials stood at 6.5%. In the control study, we filtered 5.5% of trials outside the 200-1000 ms window. The error rate in catch trials was 9.2%, and a participant was discarded for excessive errors (over 50%).

Our primary hypotheses were tested using mixed-effect linear models with reaction times as the dependent variable to check for the presence of the classical cue validity effect in the Posner cueing task and to test for the presence of the near-hand effect. For all the models, a random effect was applied on the intercept at the participant level to control for between-subject variability in reaction times distributions and model intra-subject correlation of repeated measurements. The variance-covariance matrix of all models was estimated using a robust variance estimator^67^^,^^68^^,^^69^ to obtain heteroskedasticity robust standard errors clustered at the subject level. The reported results of the mixed-effects models include the regression coefficient (B), the standardized coefficient (β), the z-statistics (z), and the p-value (α<0.05).

The main effect of our interest consisted of the impact of hand congruency (congruent, incongruent), hand presence (present, not present), and their interaction on participants’ reaction times. Specifically, the interaction between hand congruency and hand presence expresses the near-hand effect (NHE), i.e., the hypothesized decrease in reaction times when (i) the hand is present near the screen and (ii) its position is consistent with the target location in the Posner task.

To validate our results with a different statistical approach, we computed the NHE score (see Equations 1 and 2) as the difference between the mean RT of hand incongruent minus hand congruent trials. In the NH condition, we refer to congruent and incongruent trials considering the target location next to where the hand would be in the RH/SH condition, even though no hand is presented near the screen during these trials. The NHE defines a faster RT when a target appears near the robot’s hand than when no hand is near the screen. The NHE score was compared with 0 using a One-sample Wilcoxon signed-rank test. The non-parametric test was two-tailed, with an alpha level of 0.05, to determine statistical significance.

Additional analyses examined the correlations between the NHE score and 1) kinematic indexes extracted from the joint sawing task and 2) questionnaires assessing user perceptions and impressions of the robot. These analyses provided further insights into the interpretation of the main findings.

In the control study, we added the hand identity condition (self, robot) to the mixed-effect linear model used for the main analysis. The three-way interaction between hand congruency, hand presence, and hand identity expresses a difference in the emergence of the NHE when participants perform the task either with their own hand or the robot’s hand on the screen. Finally, we computed the NHE scores in the two hand identity conditions using Equation 2 ($NHE_{S}=\Delta RT_{SH}-\Delta RT_{NH}$, $NHE_{R}=\Delta RT_{RH}-\Delta RT_{NH}$). We ran One-sample Wilcoxon signed-rank tests against 0 for $NHE_{S}$ and $NHE_{R}$ and directly compared them using a Wilcoxon signed-rank test.

#### Sample size

As explained in the previous paragraph, the sample size was determined based on the principal analysis planned to test the NHE based on mixed-effect modeling. The target sample size was determined following an ad hoc simulation analysis of reaction time data using mixed-effects models.^70^ The simulation results recommend using at least 1600 observations (N. subjects∗N. trials) per condition to achieve ideal statistical power, even assuming a small effect size (d=.1). Thus, we collected data from 30 subjects who faced 54 trials for each combination of statistically relevant factors (hand congruency, hand presence). For instance, each participant performed 54 trials with the hand-congruent robot’s hand near the screen, which guaranteed the use of 1620 observations of relevant (non-catch) trials per combination.

As for the correlation analyses, we did not conduct an *a priori* power analysis. Nonetheless, we highlight that assuming a medium effect size of r = 0.5, which is comparable to the effects observed in our data, a two-tailed test with α=0.05 and power = 0.80 would require a sample size of 29 participants. This is well aligned with the sample size determined by our initial power analysis for the main effects, reinforcing the robustness of our approach. Thus, our sample size remains appropriate for detecting meaningful effects in both the primary and additional analyses.

In the control study, the analysis also included hand identity conditions (self, robot). We collected data from 22 subjects who faced 108 relevant trials (i.e., non-catch) for each combination of statistically relevant factors (hand congruency, hand presence, and hand identity). The number of relevant trials per combination is doubled because each participant repeated Posner with both hands. Hence, they faced 864 statistically relevant trials, and statistical analyses were conducted on 2376 observations per condition, well above the desired target of 1600 observations per condition.
