# Supplementary material for: Collaborating with a robot biases human spatial attention
Source: iScience. 2025 Jun 2;28(7):112791. doi: 10.1016/j.isci.2025.112791 (PMC12221521; doi:10.1016/j.isci.2025.112791)
Supplement: Document S1. Figures S1–S3 [file mmc1.pdf]

iScience, Volume *28*

Supplemental information

Collaborating with a robot biases human spatial attention

Giulia Scorza Azzarà, Joshua Zonca, Francesco Rea, Joo-Hyun Song, and Alessandra Sciutti

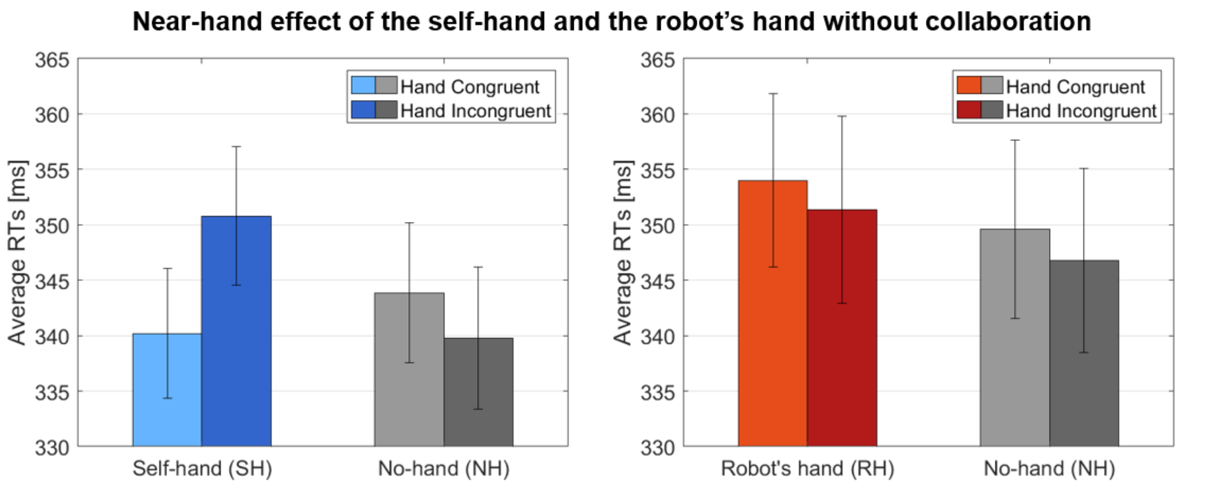

Figure S1. Average reaction times across Posner conditions without human-robot collaboration. The bar plots show the average RTs across conditions during the Posner cueing task. The blue bars refer to the self-hand (SH) condition, the red bars refer to the robot's hand (RH) condition, and the grey bars refer to the no-hand (NH) condition, with light bars representing hand-congruent trials and dark bars representing hand-incongruent trials. The error bars represent the between-subjects standard error of the means. In these plots, we observe the key results of our control study: faster RTs in the hand-congruent SH condition (left plot) and slower RTs in the hand-congruent RH condition (right plot), leading to NHE of the self-hand but no NHE of the robot's hand without human-robot collaboration. Additionally, average RTs in the NH condition remain consistent across conditions.

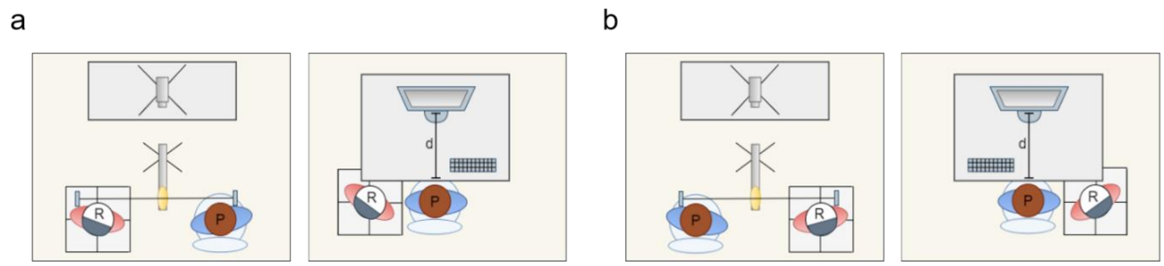

Figure S2. Experimental setup for the joint sawing task and the Posner cueing task.
(a) On the left, the participant keeps the handle in the right hand, whereas the robot uses its left hand to cut the soap; the interaction is recorded with a video camera. On the right, the participant responded to visual stimuli by pressing the space bar with the right hand while the robot sat on the left side.
(b) The setup is mirrored, so the participant used the left hand to cut the soap and respond to visual stimuli while the robot sat on the right side and used the right hand throughout the experiment.

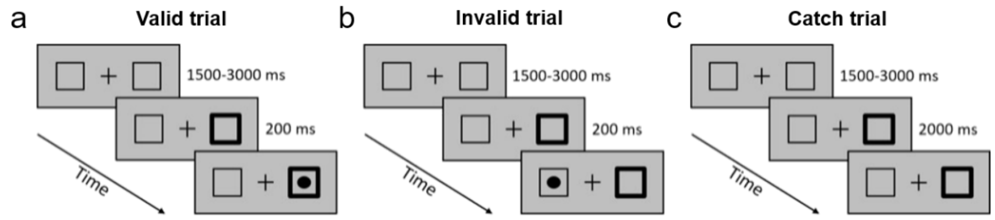

Figure S3. Functioning and trial classification of the Posner cueing task.
(a) Valid trial if the target appears in the cued square.
(b) Invalid trial if the target appears in the not cued square.
(c) Catch trial if the target does not appear.
